# Efficacy and Safety of Biological Agents in Giant Cell Arteritis: An Updated Meta-Analysis

**DOI:** 10.1055/s-0045-1809621

**Published:** 2025-06-19

**Authors:** Abdul Haseeb, Fabiha Athar, Hussain Abbas, Najia Sadiq, Faiza Naz, Erum Siddiqui, Osaid Ahmed, Umer Wamiq, Syed Ahmed Abbas Wasi, Hafsa Shuja, Bilal Aheed, Muhammad Ashir Shafique, Amna Sohail

**Affiliations:** 1 Department of Medicine, Jinnah Sindh Medical University, Karachi, Pakistan; 2 Department of Biochemistry, Jinnah Sindh Medical University, Karachi, Pakistan; 3 Department of Medicine, Jinnah Medical and Dental College, Karachi, Pakistan

**Keywords:** giant cell arteritis, biological agent, efficacy, remission, adverse effect, meta-analysis

## Abstract

**Background:**

Giant cell arteritis (GCA), impacting individuals over 50, causes vision loss, headaches, and jaw pain due to inflammation from proinflammatory cytokines and growth factors. Standard treatment involves glucocorticoids, with tocilizumab and tumor necrosis factor (TNF) inhibitors currently being studied.

**Method:**

This meta-analysis, following the Preferred Reporting Items for Systematic Reviews and Meta-Analyses and Meta-analysis of Observational Studies in Epidemiology guidelines, included adult GCA patients treated with biological agents. The search covered PubMed, Medline, Embase, and Scopus until October 2023, excluding nonhuman, pediatric, non-English, and nonrandomized studies. Data were analyzed using Review Manager 5.4, with random effects models calculating odds ratios (ORs) and 95% confidence intervals (CIs).

**Result:**

A meta-analysis of 11 studies ( *n* = 924) demonstrated higher remission rates with biological agents (OR = 2.58, 95% CI: 1.17–5.71; *p* = 0.02; *I* ^2^ = 70%), especially tocilizumab (OR = 4.30, 95% CI: 1.22–15.21; *p* = 0.02). Nonsignificant trends favored biological agents for relapse rates (OR = 0.52, 95% CI: 0.26–1.05; *p* = 0.07) and control for adverse effects (OR = 0.70, 95% CI: 0.49–1.02; *p* = 0.07). However, TNF inhibitors were linked to increased infection rates (OR = 2.41, 95% CI: 1.17–4.96; *p* = 0.02).

**Conclusion:**

Tocilizumab effectively induces remission in GCA patients, while abatacept and TNF inhibitors offer minimal benefits with increased infection risks, according to this meta-analysis. Treatment decisions should consider these factors, and larger studies are necessary to evaluate the safety and efficacy of biological agents in managing GCA.

## Introduction

Giant cell arteritis (GCA) is commonly defined as an autoimmune, inflammatory disorder that affects the tiny and medium-sized blood vessels. 1 Individuals who are vulnerable to this condition tend to be over the age of 50. The symptoms associated with this vascular disease include vision loss, headaches, and claudication of the jaw, arms, and legs. Additionally, it is worth noting that there are several conditions that are highly correlated with GCA, such as polymyalgia rheumatica, stroke, and aortic aneurysm. 2 Owing to the presence of complications such as ischemic ophthalmic, GCA is typically considered a medical emergency. 3 A range of inflammatory cells, such as proinflammatory cytokines like interleukin 1 (IL-1β), IL-6, tumor necrosis factor (TNF-α), interferon-γ, chemokine ligand 2, and adhesion molecules (e.g., E-selectin, intercellular adhesion molecule-1, vascular cell adhesion molecule-1) and growth factors (vascular endothelial growth factor, transforming growth factor β, platelet-derived growth factor), play a role in causing vascular inflammation, injury, tissue repair, and remodeling. 4

GCA is typically managed through various therapeutic approaches; however, glucocorticoids are the preferred treatment option. 5 Predicting the duration of glucocorticoid therapy for patients with GCA based on their initial symptoms has proven challenging. 6 A biological agent is any bacteria, virus, protozoan, parasite, fungus, or toxin that can be deliberately employed as a weapon in bioterrorism or biological warfare. 7 In addition to these live or reproducing infections, bioagents include poisons and biotoxins. More than 1,200 types of potentially weaponizable bioagents have been described and analyzed to date. Biological therapies, such as tocilizumab, adalimumab, etanercept, infliximab, abatacept, and TNF inhibitor, have been studied as alternative medicines to treat GCA, but their function and effectiveness remain uncertain. 8

Giant cells and macrophages of the intima and media of inflamed arteries express TNF, which suggests that TNF may be involved in mediating the infiltration and arterial wall breakdown characteristics of GCA. 9 Recent randomized controlled trials (RCTs) investigating the efficacy of anti-TNFα medications in patients newly diagnosed with GCA have yielded inconsistent results. One study examined the impact of supplementing prednisolone with a 10-week course of adalimumab in this patient population. The findings indicated no significant increase in the number of patients achieving remission while maintaining corticosteroid doses below 0.1 mg/kg at the 6-month evaluation point. 10 This underscores the need for careful assessment of the efficacy of adjunct therapies in the treatment of GCA. Several clinical studies have been conducted to determine the safety and efficacy of biological therapies for the management of GCA. 11 To effectively select the most suitable biological agent based on a patient's condition, it is important to evaluate and compare both the efficacy and adverse effects of various biological agents. 12 13 14 This meta-analysis comprehensively examined and contrasted the utilization of various biological agents and their efficacy and adverse effects in patients with GCA.

## Methods and Materials

### Study Protocol

This systematic review and meta-analysis was performed in accordance with the Preferred Reporting Items for Systematic Reviews and Meta-Analyses (PRISMA) and Meta-analysis of Observational Studies in Epidemiology (MOOSE) guidelines. The present systematic review and meta-analysis has been registered on the PROSPERO database (CRD42023471144).

### Literature Search and Strategy

A comprehensive literature search was conducted for this study using several electronic databases, including PubMed, Medline, Embase, and Scopus from inception to October 2023. These databases were used to identify relevant studies that examined the safety and efficacy of biological agents in patients with GCA.

The search strategy included Medical Subject Headings terms and keywords such as “Giant cell arteritis,” “GCA,” “biological agents,” “tocilizumab,” “abatacept,” “etanercept,” “adalimumab,” “infliximab,” and “TNF inhibitor.” The detailed breakdown of the search strings is provided in Supplementary Material S1 (online only). Moreover, only English language articles obtained from the search were utilized. Additionally, reference lists of included studies were reviewed to identify additional relevant research.

### Eligibility Criteria

#### Inclusion Criteria

Research studies that fulfilled the following criteria were considered eligible for inclusion: (1) adult patients (18 years or older) with a confirmed diagnosis of GCA; (2) patients receiving treatment with biological agents (tocilizumab, abatacept, etanercept, adalimumab, infliximab, TNF inhibitor); (3) a control group comprising patients who received standard therapy or a placebo; (4) studies having primary outcomes of clinical efficacy through remission and relapse, and safety profile through adverse effects and infection outcome following biological agents in patients with GCA; and (5) RCTs, observational studies, and cohort studies satisfying the above eligibility criteria.

Efficacy outcomes included the number of patients who achieved renal remission and the number of patients who experienced relapse during the follow-up period, while the safety outcomes included the number of serious adverse events (SAEs) and incidence of infection. Remission and relapse were defined in accordance with the definitions used in the original studies.

#### Exclusion Criteria

We excluded the studies if they: (1) involved nonhuman participants; (2) were conducted in languages other than English; (3) were editorials, case–control studies, case series, and single-arm studies; (4) did not produce the anticipated outcomes; and (5) involved pediatric populations.

### Study Screening and Extraction

Studies were initially screened by reviewing their titles and abstracts. Subsequently, a full text review of the selected studies was performed. Two reviewers conducted the data screening, and any discrepancies were resolved through mutual consultation with a third reviewer. The name of the first authors, the year of publication, and the baseline information, including demographic data (population age and sample size), intervention details (duration and dosage of biological agents), and outcome data (efficacy and safety), were extracted. Efficacy outcomes were determined through the assessment of relapse and remission, which included the number of patients who achieved renal remission and the number of patients who experienced relapse during the follow-up period. Remission and relapse were defined according to the criteria used in the original studies. The safety outcomes included the incidence of infection and the occurrence of SAEs. 3

### Data Analysis and Quality Assessment

The data was analyzed by a single author using Review Manager 5.4 (Cochrane collaboration) software. A random effects model was applied, and odds ratios (ORs) with 95% confidence intervals (CIs) were calculated for dichotomous outcomes. The *I* ^2^ statistic was used to measure heterogeneity between trials, with values of 25%, 25% to 50%, and over 50% indicating modest, moderate, and significant heterogeneity, respectively. Forest plots were generated for adverse effects, relapse, remission, and infection outcomes, with OR effect sizes and 95% CIs. The evaluation of the risk of bias in the included RCTs was done using the Cochrane tool for risk of bias. The categorization of the studies was based on the assessment of selection bias, reporting bias, other prejudices, and performance bias, which resulted in the classification of studies as having low, high, or uncertain risk. Additionally, the Newcastle–Ottawa Scale (NOS) was applied to assess the risk of bias in cohorts and observational studies, which was implemented alongside the Cochrane tool, see Supplementary Table S1 (online only).

## Result

### Study Characteristics

**Fig. 1:**
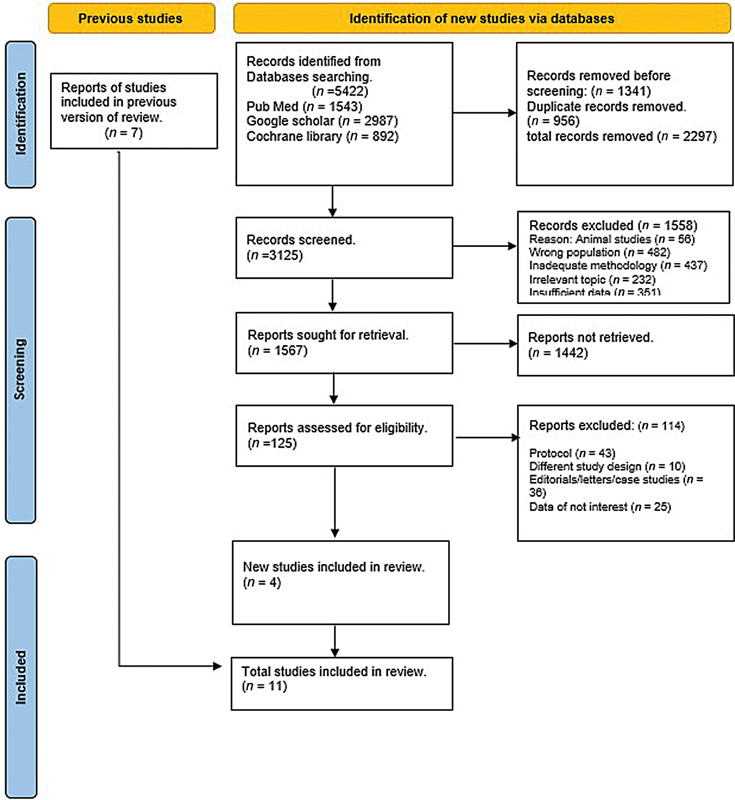
Preferred Reporting Items for Systematic Reviews and Meta-Analyses (PRISMA) flowchart representing the process of selection of included studies.

The systematic literature search using various databases yielded a total of 5,422 articles. After removing duplicates, 3,125 articles underwent screening. Of these, 1,567 articles were screened primarily based on their titles and abstracts, leading to the identification of 125 articles for thorough screening. Secondary screening resulted in the exclusion of 114 articles due to irrelevant data and failure to meet the predefined inclusion criteria. Ultimately, 11 articles were included in our meta-analysis. The PRISMA flowchart depicting the literature selection process is presented in Fig. 1 . The baseline characteristics of the included studies are summarized in Table 1 . The eligible studies comprised a sample population of 924 patients diagnosed with GCA, with a median age of 70 to 79 years. Out of the 11 studies, 5 were conducted in the United States, 4 were conducted in Europe (one each in Italy, Germany, France, and Spain), and only 1 was conducted in the United Kingdom. The median follow-up ranged from 1.4 to 19.2 months. All studies utilized interventions involving biological agents, such as tocilizumab, adalimumab, etanercept, infliximab, and abatacept, as the intervention group, with placebo as the control group.

**Table 1:** Baseline characteristic table of included studies

Author	Year	Design	Intervention (biological agents) Male:Females	Control (placebo) Male:Females	Population	Outcome
Stone et al	2022	RCT	100 22:78	10126:75	Patients with new-onset or relapsing GCA received blinded tocilizumab weekly or every other week or a placebo for 52 weeks	TCZ QW delayed duration of flare and decreased the cumulative glucocorticoid dose
Speira et al 25	2021	RCT	60 14:46	44 6:38	GCA population was administered TCZ every 2 weeks and a placebo for 26 or 52 weeks, along with a prednisone taper	TCZ improved clinical outcomes in patients who presented with PMR symptoms only, cranial symptoms only, or both at baseline
Strand et al 26	2019	RCT	10022:78	5114:37	Subcutaneous TCZ 162 mg administered to GCA population in conjunction with a 26-week prednisone taper or a placebo along with a 26-week or 52-week prednisone taper	SF-36 and FACIT-Fatigue scales improved more significantly for those receiving the combination therapy as compared with those who were only given prednisone
Nannini et al 27	2019	Prospective study	6 2:4	9 2:7	GCA population receiving intravenous TCZ 8 mg/kg for 4 weeks PDN 5 mg/kg for 4 weeks	TCZ therapy was well tolerated with no severe adverse events as compared with PDN
Stone-1 et al	2017	RCT	100 22:78	51 14:37	The GCA population received 162 mg of subcutaneous tocilizumab weekly for 52 weeks	At week 52, 56% of patients on tocilizumab achieved sustained remission compared with 14% in the placebo group
Stone-2 et al	2017	RCT	50 15:35	50 12:38	GCA population received subcutaneous tocilizumab at 162 mg, every other week for 52 weeks	At week 52, 53% of those treated with tocilizumab every other week achieved sustained remission, compared with only 14% in the placebo group
Villiger and colleagues	2019	RCT	20 7:13	10 2:8	Patients with GCA received 8 mg/kg of TCZ intravenously every 4 weeks for 52 weeks	Tocilizumab group achieved 85% relapse-free survival, while placebo group achieved 20%
Seror et al	2014	RCT	34 10:24	36 8:28	GCA population received 10 weeks subcutaneous adalimumab 40 mg every other week	The percentage of patients achieving remission was 58.9% in the adalimumab arm and 50.0% in the placebo arm
Martinez-Taboada et al 28	2008	RCT	8	9	Administered etanercept at 25 mg twice weekly via subcutaneous injection to the GCA population	50% of patients in the etanercept group and 22.2% in the placebo group were able to manage their condition without corticosteroid treatment
Hoffman et al 29	2007	RCT	28	16	GCA population received infliximab, 5 mg/kg, or placebo at 0, 2, and 6 weeks and every 8 weeks thereafter	Infliximab therapy did not increase the proportion of patients without relapse compared with placebo (43% vs. 50%) (NS)
Langford et al	2017	RCT	20	21	Abatacept dosage for GCA patients: 10 mg/kg (max 1000 mg), IV infusion on days 1, 15, and 29, 8 weeks, and then every 4 weeks	Abatacept-treated patients had a 12-month relapse-free survival rate of 48%, compared with 31% for those receiving a placebo

Abbreviations: FACIT, Functional Assessment of Chronic Illness Therapy-Fatigue; GCA, giant cell arteritis; IV, intravenous; NS, nonsignificant; PDN, prednisolone; PMR, polymyalgia rheumatic; QW, subcutaneously weekly; RCT, randomized controlled trial; SF-36, 36-Item Short Form Survey; TCZ, tocilizumab.

### Primary Outcomes

#### Remission

**Fig. 2:**
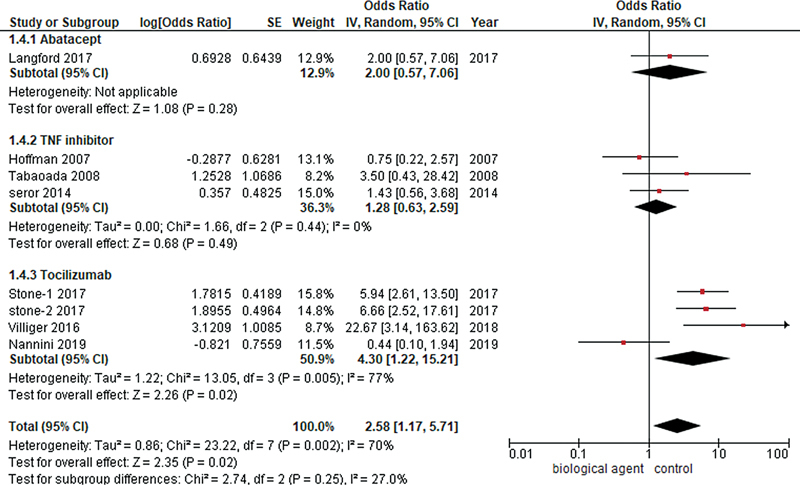
Forest plot demonstrating remission outcome of giant cell arteritis among different biological agents versus control group.

The phase during which there are no clinical manifestations of the disease is referred to as remission. 15 The results of a pooled analysis of eight studies comparing biological agents to the control group demonstrated that the overall remission rate was significantly higher in the biological agent group than in the control group (OR = 2.58, 95% CI: 1.17–5.71; *p* -value = 0.02; *I* ^2^ = 70%). A subgroup assessment was conducted to determine the remission rate for different biological agents, namely, tocilizumab, abatacept, and TNF inhibitors. The ORs and their associated values for each comparison are as follows: the subgroup analysis of abatacept showed a trend toward increased odds of remission in the abatacept group compared with the control group, although the result was statistically insignificant (OR = 2.00, 95% CI: 0.57–7.06; *p* = 0.28). Similarly, the subgroup analysis of three studies of TNF inhibitors determined increased odds of remission with the use of TNF inhibitors compared with the control group, although the result was statistically insignificant (OR = 1.28; 95% CI: 0.63–2.59; *I* ^2^ = 0%; *p* = 0.49). Among all biological agents, tocilizumab was found to have the highest odds of remission in the GCA population (OR = 4.30, 95% CI: 1.22–15.21; *I* ^2^ = 77%; *p* = 0.02), as illustrated in Fig. 2 .

#### Relapse

**Fig. 3:**
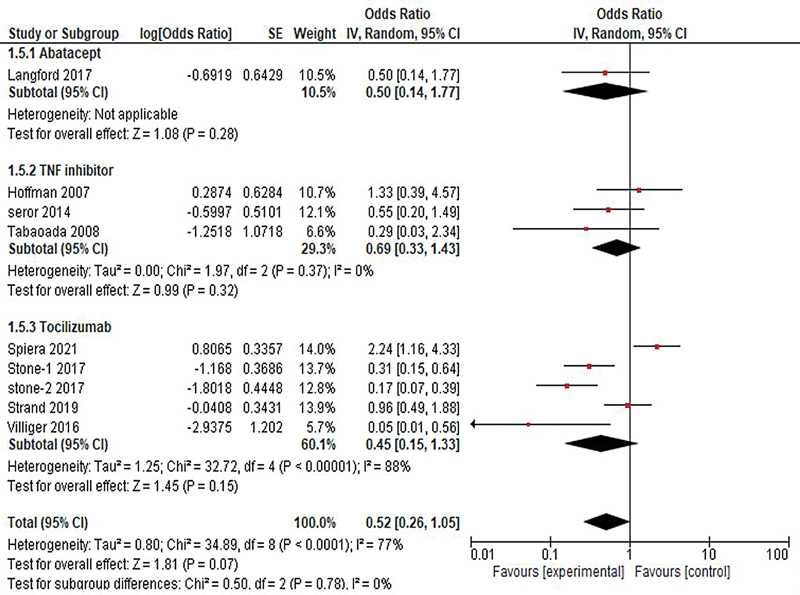
Forest plot representing relapse outcome of giant cell arteritis among different biological agents versus control.

The findings from nine studies revealed a general trend of lower odds of relapse among biological agents compared with the control group, although the difference was not statistically significant (OR = 0.52, 95% CI: 0.26–1.05; *p* = 0.07; *I* ^2^ = 77%). To further investigate the efficacy of different biological agents in treating the outcome of relapse of GCA, a subgroup analysis was performed. The analysis of the abatacept group showed a potential decrease in the odds of relapse, although the result was not statistically significant (OR = 0.50, 95% CI: 0.57, 0.14–1.77; *p* = 0.28). Similarly, the TNF inhibitors group revealed a slight decrease in the odds of experiencing relapse (OR = 0.69, 95% CI: 0.33–1.43; *I* ^2^ = 0%; *p* = 0.32), and so did the tocilizumab group, although the result was not statistically significant (OR = 0.45, 95% CI: 0.15–1.33; *I* ^2^ = 88%; *p* = 0.15), as shown in Fig. 3 .

**Fig. 4:**
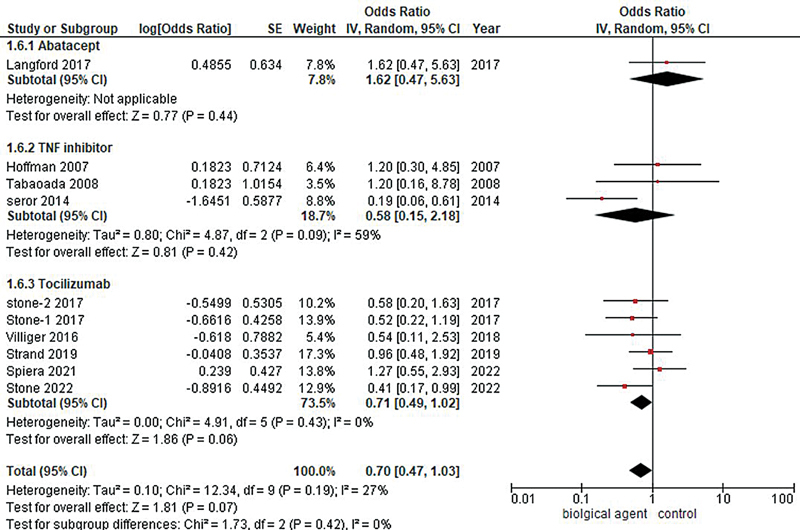
Forest plot showing adverse effect profile of different biological agents versus control.

### Secondary Outcomes

#### Adverse Effect Profile

**Fig. 5:**
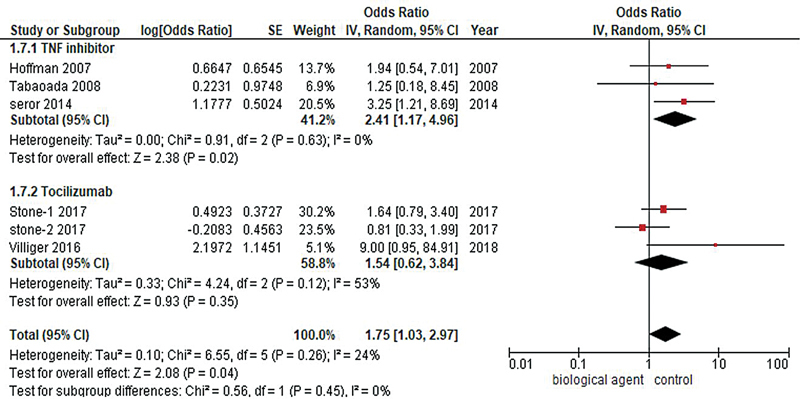
Forest plot demonstrating infection outcome among different biological agents versus control.

The pooled analysis of 10 studies revealed a statistically nonsignificant result, showing a decrease in the odds of experiencing an adverse effect profile in patients treated with biological agents compared with the control group (OR = 0.70, 95% CI: 0.49–1.02; *p* -value = 0.07; *I* ^2^ = 27%). By conducting a subgroup analysis, adverse effects among different biological agents were assessed, which revealed an increase in the odds of adverse effect profile among the abatacept group, although statistically nonsignificant (OR = 1.62, 95% CI: 0.47–5.63; *p* -value = 0.44). However, subgroup analysis showed a decrease in the odds of experiencing an adverse effect profile among TNF inhibitors (OR = 0.58, 95% CI: 0.15–2.18; *I* ^2^ = 59%; *p* -value = 0.42) and tocilizumab (OR = 0.71, 95% CI: 0.49–1.02; *I* ^2^ = 0%; *p* -value = 0.06), although these results were statistically insignificant, see Figs. 4 and 5 .

#### Infection

Analysis of six studies revealed that biological agents were linked to a statistically nonsignificant increase in the incidence of infection rates when compared with patients in the placebo group (OR = 1.75, 95% CI: 1.03–2.97; *p* -value = 0.07; *I* ^2^ = 27%). Moreover, the subgroup analysis indicated statistically significant higher incidences of infection with TNF inhibitors (OR = 2.41, 95% CI: 1.17–4.96; *I* ^2^ = 0%; *p* = 0.02) and tocilizumab (OR: 1.54, 95% CI: 0.62–3.84; *I* ^2^ = 53%; *p* = 0.35), as shown in Fig. 5 .

**Fig. 6:**
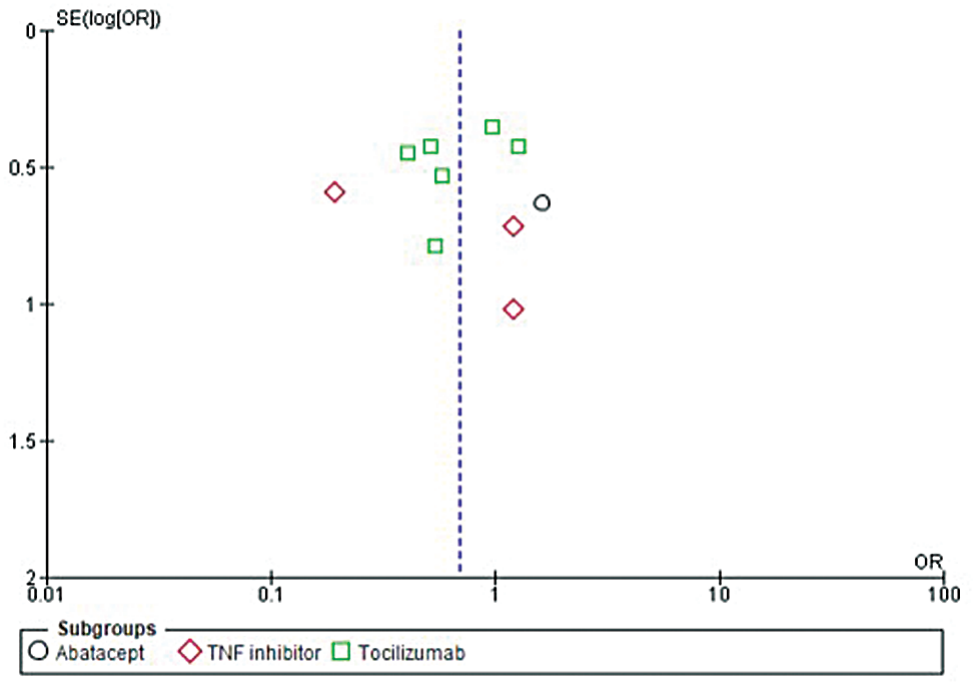
Funnel plot demonstrating publication bias.

**Fig. 7:**
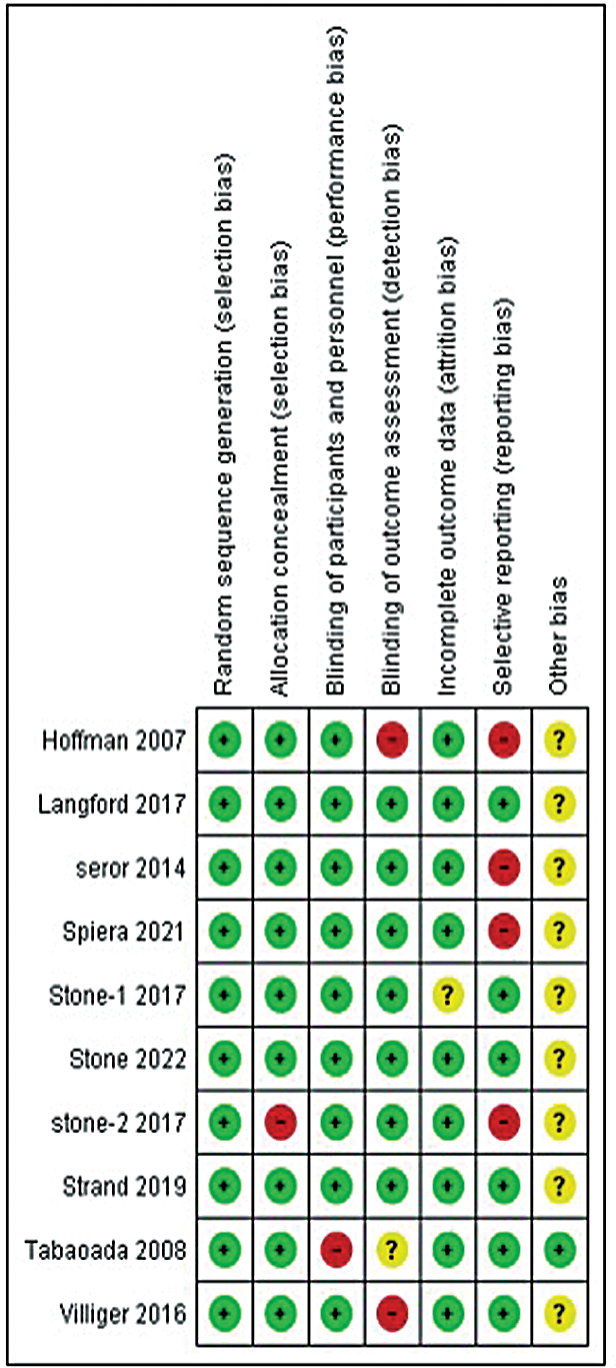
Cochrane tool for risk of bias of included randomized controlled trials (RCTs).

The evaluation of publication bias for the studies included in this research was performed using a funnel plot, as shown in Fig. 6 . The risk of bias associated with the RCTs included in this study was assessed using the Cochrane method for risk of bias assessment. Four out of the 10 RCTs had reporting bias, but the majority of the studies were deemed to be at low risk for various biases, including selection, performance, and attrition, as shown in Fig. 7 . The NOS was employed to evaluate the risk of bias in the observational study that was part of the meta-analysis. This scale assesses exposure, comparability, and selection, and the NOS scores for the studies ranged from 6 to 9, indicating generally high quality across trials. The observational study included was rated as good quality, with a NOS score between 7 and 9, see Supplementary Table S1 (online only).

## Discussion

The management of large vessel vasculitis (LVV), as outlined by the 2018 EULAR recommendations, emphasizes personalized treatment strategies. The initial approach to induce remission in cases of active GCA or Takayasu arteritis (TAK) involves administering high-dose glucocorticoid therapy, specifically 40 to 60 mg/day of prednisolone-equivalent. Tocilizumab is advised as an adjunctive therapy for patients with GCA who exhibit refractory disease, experience relapses, or are prone to adverse effects from glucocorticoids, with methotrexate serving as a potential alternative. For patients with refractory or recurrent conditions, the consideration of biologic supplements is essential; however, in the treatment of TAK, nonbiologic glucocorticoid-sparing medications should be used in conjunction with glucocorticoids. The routine use of antiplatelet or anticoagulant medication for LVV is no longer recommended unless indicated for other reasons. 16

The findings of our meta-analysis offer illuminating insights into the efficacy and safety of biological agents in treating GCA. Our analysis encompassed a substantial body of literature comprising 11 relevant studies, encompassing a total of 924 patients with GCA. The age range of the study population was between 70 and 79 years, with various biological agents employed as interventions in the included studies. A key discovery highlighted in our research pertains to remission rates. The study conducted by Song et al demonstrated that biological agents, such as tocilizumab, were more effective in achieving remission in patients with GCA compared with control groups. Additionally, the study indicates that TNF inhibitors and abatacept did not exhibit a significant difference in remission rates when compared with placebo. 3 This finding confirms that biological agents like tocilizumab are effective in improving remission rates in GCA patients compared with control groups. In a recent study by Stone et al, it is demonstrated that tocilizumab when administered weekly significantly delays the time to flare and reduces the cumulative glucocorticoid dose in patients with relapsing and new-onset GCA. These findings support the initiation of biological agents preferably tocilizumab, as a first-line therapeutic option for all patients presenting with active GCA. 17 Furthermore, subsequent investigations have called into question earlier findings, confirming that tocilizumab is more effective than placebo in achieving remission and preventing relapse in GCA patients. The results of the GiACTA trial further bolster this evidence, showing that tocilizumab not only improves remission rates but also reduces the need for glucocorticoids in both new-onset and relapsing GCA cases. Together, these studies highlight the critical role of tocilizumab in the management of GCA, reinforcing its position as a preferred treatment strategy.

Our findings on relapse rates indicated a nonsignificant result between biological agents and placebo, although there was a trend suggesting a decrease in the odds of relapse among patients treated with biological agents. However, the study conducted by Villiger and colleagues found no clinical or imaging variables that could predict relapse in patients with GCA who were treated with tocilizumab. 7 Considerable heterogeneity was observed in the results concerning remission and relapse, with the tocilizumab group exhibiting greater heterogeneity in the risk of relapse ( *I* ^2^ = 88%). This pronounced heterogeneity is likely attributable to variations in patient characteristics and the inconsistent definitions of remission and relapse across studies. Each study included in this meta-analysis employed different criteria to assess the risk of relapse, which may have contributed to the observed variability. Standardizing these definitions in future research could enhance comparability and improve overall outcomes. The recombinant Ig-CTLA-4 fusion protein, abatacept, binds to CD80/86 on antigen-presenting or activated dendritic cells. This interaction inhibits the binding of CD28, thereby preventing the activation of CD4+ T cells and the production of IL-6. In a study conducted by Langford et al, abatacept emerged as a novel treatment for GCA, demonstrating the ability to maintain corticosteroid-free remission for an average of 9.9 months, compared with 3.9 months for patients receiving a placebo. Furthermore, individuals receiving intravenous abatacept exhibited a relapse-free survival rate of 48% at 12 months, in contrast to 31% for those receiving a placebo. 15 Our research uncovered that employing a TNF inhibitor and abatacept for the management of GCA yielded no beneficial outcomes. Both demonstrated no statistical significance in terms of remission, relapse, adverse effects, and infection rate. The precise role of TNF in the pathophysiology of GCA remains unclear, despite its abundant presence in biopsy samples showcasing vascular damage resulting from the condition. 18 In our meta-analysis, while abatacept was associated with a significantly lower incidence of infections in patients with GCA, we concur with the conclusions of Song et al, which determined that neither abatacept nor TNF inhibitors exhibited significant efficacy in achieving remission or preventing relapse compared with placebo. Specifically, their study indicated that TNF inhibitors were associated with an increased risk of infections. These findings underscore the complexity of evaluating treatment options in GCA, where certain agents may mitigate adverse effects but lack overall effectiveness in managing the disease. 3 Patients with GCA who exhibited an inflammatory response showed heightened tissue expression of TNF-α, and this increased production of TNF-α was correlated with increased steroid requirements and recurrent disease. 19 The lack of efficacy of TNF inhibitors might be attributed to the different mechanisms of action, as IL-6 inhibition using tocilizumab was more successful. 20 The preferred treatment method for GCA is high-dose corticosteroid therapy, which has proven to be effective. However, it is essential to be informed of the potential side effects associated with long-term corticosteroid use. Prolonged corticosteroid therapy has been linked to several common and potentially harmful side effects, such as diabetes mellitus, vertebral compression fractures, steroid myopathy, steroid psychosis, and an increased risk of infections due to immunosuppression. 21 It is important to emphasize that individuals with GCA, particularly those using tocilizumab and large doses of prednisone, often have flare-ups of their disease. 22 This emphasizes the need to manage GCA carefully to avoid overtreating the condition. It also emphasizes how critical it is to identify potential targets for biological therapies that can more effectively control disease activity and diminish the incidence of flare-ups as comprehensively as feasible. 23 24

### Limitations

This meta-analysis has several limitations. Larger-scale studies are required, particularly for the treatment of GCA with abatacept and TNF inhibitors, as insufficient patient data makes it difficult to draw firm conclusions. First, low sensitivity and possible publication bias might have been caused by the comparatively limited number of included studies. Second, the studies might have been heterogeneous due to variations in follow-up duration, study design, and demographics, which could have affected the results. This variation was further enhanced by various definitions of infection and recurrence. Additionally, by focusing only on safety—as measured by the frequency of infections and side effects—and efficacy—as demonstrated by the rates of remission and relapse—the analysis disregarded other important outcomes.

## Conclusion

This research examines various efficacy and safety profiles of biological agents for treating GCA. Tocilizumab has demonstrated a considerable improvement in remission rates compared with a placebo, while abatacept and TNF inhibitors did not show significant advantages in achieving or preventing relapses. Although overall adverse effects were not significantly increased with the use of biological agents, TNF inhibitors were linked to a higher infection rate. These results emphasize the potential of tocilizumab in GCA management, while also emphasizing the need for further research to evaluate the effectiveness and safety of other biological agents. Thus, treatment decisions should consider individual patient profiles weighing both the therapeutic benefits and potential risks. Additional extensive studies are necessary to reach conclusions about the efficacy of biological agents in the near future.

## References

[1] Yates M Loke Y K Watts R A MacGregor A J Prednisolone combined with adjunctive immunosuppression is not superior to prednisolone alone in terms of efficacy and safety in giant cell arteritis: meta-analysis Clin Rheumatol 2014 33 02 227 236 24026674 10.1007/s10067-013-2384-2

[2] Salvarani C Cantini F Boiardi L Hunder G G Polymyalgia rheumatica and giant-cell arteritis N Engl J Med 2002 347 04 261 271 12140303 10.1056/NEJMra011913

[3] Song G G Lee Y H Efficacy and safety of biological agents in patients with giant cell arteritis: a meta-analysis of randomized trials Int J Clin Pharmacol Ther 2020 58 09 504 510 32567545 10.5414/CP203738

[4] Visvanathan S Rahman M U Hoffman G S Tissue and serum markers of inflammation during the follow-up of patients with giant-cell arteritis–a prospective longitudinal study Rheumatology (Oxford) 2011 50 11 2061 2070 21873264 10.1093/rheumatology/ker163 PMC3198905

[5] Proven A Gabriel S E Orces C O'Fallon W M Hunder G G Glucocorticoid therapy in giant cell arteritis: duration and adverse outcomes Arthritis Rheum 2003 49 05 703 708 14558057 10.1002/art.11388

[6] Gabriel S E O'Fallon W M Achkar A A Lie J T Hunder G G The use of clinical characteristics to predict the results of temporal artery biopsy among patients with suspected giant cell arteritis J Rheumatol 1995 22 01 93 96 7699690

[7] Adler S Reichenbach S Gloor A Yerly D Cullmann J L Villiger P M Risk of relapse after discontinuation of tocilizumab therapy in giant cell arteritis Rheumatology (Oxford) 2019 58 09 1639 1643 30915462 10.1093/rheumatology/kez091

[8] Osman M Pagnoux C Dryden D M Storie D Yacyshyn E The role of biological agents in the management of large vessel vasculitis (LVV): a systematic review and meta-analysis PLoS One 2014 9 12 e115026 25517966 10.1371/journal.pone.0115026 PMC4269410

[9] Airò P Antonioli C M Vianelli M Toniati P Anti-tumour necrosis factor treatment with infliximab in a case of giant cell arteritis resistant to steroid and immunosuppressive drugs Rheumatology (Oxford) 2002 41 03 347 349 11934977 10.1093/rheumatology/41.3.347

[10] Seror R Baron G Hachulla E Adalimumab for steroid sparing in patients with giant-cell arteritis: results of a multicentre randomised controlled trial Ann Rheum Dis 2014 73 12 2074 2081 23897775 10.1136/annrheumdis-2013-203586

[11] Field M Cook A Gallagher G Immuno-localisation of tumour necrosis factor and its receptors in temporal arteritis Rheumatol Int 1997 17 03 113 118 9352606 10.1007/s002960050019

[12] Lee Y H Association between the neutrophil-to-lymphocyte ratio, and platelet-to-lymphocyte ratio and rheumatoid arthritis and their correlations with the disease activity: a meta-analysis J Rheum Dis 2018 25 03 169 178

[13] Lee Y H Song G G Causal association between rheumatoid arthritis with the increased risk of type 2 diabetes: a Mendelian randomization analysis J Rheum Dis 2019 26 02 131 136

[14] Lee Y H Meta-analysis of genetic association studies Ann Lab Med 2015 35 03 283 287 25932435 10.3343/alm.2015.35.3.283 PMC4390695

[15] Vasculitis Clinical Research Consortium Langford C A Cuthbertson D Ytterberg S R A randomized, double-blind trial of abatacept (CTLA-4Ig) for the treatment of giant cell arteritis Arthritis Rheumatol 2017 69 04 837 845 28133925 10.1002/art.40044 PMC5378642

[16] Hellmich B Agueda A Monti S 2018 Update of the EULAR recommendations for the management of large vessel vasculitis Ann Rheum Dis 2020 79 01 19 30 31270110 10.1136/annrheumdis-2019-215672

[17] Stone J H Spotswood H Unizony S H New-onset versus relapsing giant cell arteritis treated with tocilizumab: 3-year results from a randomized controlled trial and extension Rheumatology (Oxford) 2022 61 07 2915 2922 34718434 10.1093/rheumatology/keab780 PMC9258533

[18] Greigert H Genet C Ramon A Bonnotte B Samson M New insights into the pathogenesis of giant cell arteritis: mechanisms involved in maintaining vascular inflammation J Clin Med 2022 11 10 2905 35629030 10.3390/jcm11102905 PMC9143803

[19] González-Gay MÁ Pina T Prieto-Peña D Calderon-Goercke M Gualillo O Castañeda S Treatment of giant cell arteritis Biochem Pharmacol 2019 165 230 239 31034796 10.1016/j.bcp.2019.04.027

[20] Ogata A Kato Y Higa S Yoshizaki K IL-6 inhibitor for the treatment of rheumatoid arthritis: a comprehensive review Mod Rheumatol 2019 29 02 258 267 30427250 10.1080/14397595.2018.1546357

[21] Caselli R J Hunder G G Neurologic complications of giant cell (temporal) arteritis Semin Neurol 1994 14 04 349 353 7709085 10.1055/s-2008-1041094

[22] Stone J H Tuckwell K Dimonaco S Glucocorticoid dosages and acute-phase reactant levels at giant cell arteritis flare in a randomized trial of tocilizumab Arthritis Rheumatol 2019 71 08 1329 1338 30835950 10.1002/art.40876 PMC6772126

[23] Moiseev S Novikov P Meshkov A Smitienko I Biological agents for giant cell arteritis: treat to target Ann Rheum Dis 2016 75 09 e58 27401743 10.1136/annrheumdis-2016-210061

[24] Harrington R Al Nokhatha S A Conway R Biologic therapies for giant cell arteritis Biologics 2021 15 17 29 33442231 10.2147/BTT.S229662 PMC7797292

[25] Spiera R Unizony S H Bao M Luder Y Han J Pavlov A Stone J H Tocilizumab vs placebo for the treatment of giant cell arteritis with polymyalgia rheumatica symptoms, cranial symptoms or both in a randomized trial Semin Arthritis Rheum 2021Apr;51 02 469 476 33784598 10.1016/j.semarthrit.2021.03.006

[26] Strand V Dimonaco S Tuckwell K Klearman M Collinson N Stone J H Health-related quality of life in patients with giant cell arteritis treated with tocilizumab in a phase 3 randomised controlled trial Arthritis Res Ther 2019Feb 20;21 01 64 30786937 10.1186/s13075-019-1837-7 PMC6381622

[27] Nannini C Niccoli L Sestini S Laghai I Coppola A Cantini F Remission maintenance after tocilizumab dose-tapering and interruption in patients with giant cell arteritis: an open-label, 18-month, prospective, pilot study Ann Rheum Dis 2019Oct;78 10 1444 1446 31213436 10.1136/annrheumdis-2019-215585

[28] Martínez-Taboada V M Rodríguez-Valverde V Carreño L López-Longo J Figueroa M Belzunegui J Mola E M Bonilla G A double-blind placebo controlled trial of etanercept in patients with giant cell arteritis and corticosteroid side effects Ann Rheum Dis 2008May;67 05 625 630 18086726 10.1136/ard.2007.082115

[29] Infliximab-GCA Study Group Hoffman G S Cid M C Rendt-Zagar K E Merkel P A Weyand C M Stone J H Salvarani C Xu W Visvanathan S Rahman M U Infliximab for maintenance of glucocorticosteroid-induced remission of giant cell arteritis: a randomized trial Ann Intern Med 2007May 1;146 09 621 630 17470830 10.7326/0003-4819-146-9-200705010-00004

