# Supplementary material for: Efficacy and Safety of Biological Agents in Giant Cell Arteritis: An Updated Meta-Analysis
Source: Avicenna J Med. 2025 Jun 19;15(2):64–73. doi: 10.1055/s-0045-1809621 (PMC12178671; doi:10.1055/s-0045-1809621)
Supplement: Supplementary file 1 — Supplementary Material [file 10-1055-s-0045-1809621-s240178.pdf]

Supplementary Material S1: Search string

("giant cell arteritis"[MeSH Terms] OR ("giant"[All Fields] AND "cell"[All Fields] AND "arteritis"[All Fields]) OR "giant cell arteritis"[All Fields] OR ("giant cell arteritis"[MeSH Terms] OR ("giant"[All Fields] AND "cell"[All Fields] AND "arteritis"[All Fields]) OR "giant cell arteritis"[All Fields] OR ("temporal"[All Fields] AND "arteritis"[All Fields]) OR "temporal arteritis"[All Fields])) AND ("biological factors"[MeSH Terms] OR ("biological"[All Fields] AND "factors"[All Fields]) OR "biological factors"[All Fields] OR ("biological"[All Fields] AND "agent"[All Fields]) OR "biological agent"[All Fields]).

Supplementary Table S1 N Newcastle–Ottawa Scale (NOS) assessing bias in included observational study

First author	Newcastle–Ottawa Quality Assessment Scale									Meta-analysis	
	Selection				Compar-ability		Outcome			Eligible	selected
	1	2	3	4	5	6	7	8	9		
Nannini 2019	*	*	*	*	*	/	*	*	/	yes	yes

Newcastle–Ottawa Scale						
Newcastle–Ottawa Quality Assessment Scale						
Selection:	1	Representation of the intervention cohort (Biological agent)				
	2	Selection of the non-intervention cohort				
	3	Has the correct intervention been utilized?				
Comparability:	5	Are the cohorts comparable based on the basis of the design or analysis: age, sex and injury severity?				
	6	Are the cohorts comparable on the basis of the design or analysis? Additional factors				
Outcome:	7	Was the outcome assessed?				
	8	Was the follow up long enough for measured outcomes to occur?				
	9	Was the cohort follow up long enough?				

 © 2025. The Author(s).
